# Autumn migration of black‐necked crane (*Grus nigricollis*) on the Qinghai‐Tibetan and Yunnan‐Guizhou plateaus

**DOI:** 10.1002/ece3.10492

**Published:** 2023-09-07

**Authors:** Zhen Pu, Yumin Guo

**Affiliations:** ^1^ School of Ecology and Nature Conservation Beijing Forestry University Beijing China

**Keywords:** autumn migration, *Grus nigricollis*, migration routes, Qinghai‐Tibetan Plateau, satellite tracking, Yunnan‐Guizhou Plateau

## Abstract

Despite previous research efforts, the majority migration routes of the black‐necked cranes (*Grus nigricollis*) have remained veiled. In this study, we utilized satellite telemetry data from 45 cranes between 2015 and 2021 to unveil critical insights. Our results revealed 11 distinct autumn migration routes and one sedentary flock, of which eight routes and the sedentary flock were previously undocumented. Our findings highlighted the remarkable diversity in the migration routes of black‐necked cranes, especially in terms of migration orientations, spatial–temporal patterns, and altitudinal movement patterns. Cranes breeding on the eastern, northern, and central Qinghai‐Tibetan Plateau migrated southward, while those on the northern slopes of the Himalayas migrated eastward, westward, northward, or opted to remain sedentary. Moreover, we expanded the known range of migration distances to 84–1520 km at both ends (excluding sedentary individuals) and identified two long‐term (Da Qaidam and Chaka) and one short‐term (Gyatong grassland) stopover sites. Furthermore, our study revealed that the breeding colonies in the Qilian Mountains on the northeastern Qinghai‐Tibetan Plateau utilized long‐term stopover sites before embarking on significant altitude ascent, while other flocks displayed more urgent migration patterns, preferring to roost only at night. By unveiling the near‐complete autumn migration routes of black‐necked cranes, our research has contributed to discovering the critical habitats and connectivity among various breeding colonies, which is instrumental in developing effective seasonal conservation plans.

## Graphical abstract

The black‐necked crane is the only alpine crane species endemic to the Qinghai‐Tibetan and Yunnan‐Guizhou plateaus. We identiflied 11 distinct autumn migration routes and one sedentary flock, of which eight routes and the sedentary flock were previously undocumented. Our findings highlighted the remarkable diversity in the migration routes of black‐necked cranes, especially in terms of migration orientations, spatial–temporal patterns, and altitudinal movement patterns.

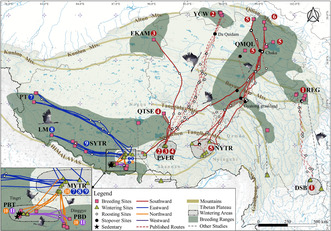

## 1 INTRODUCTION

Annual bird migrations between breeding and non‐breeding areas have long intrigued researchers (Newton, 2008). Migration has many different forms. They are often categorized by geographically oriented movements, including longitudinal (Chernetsov et al., 2008; Dufour et al., 2021), latitudinal (Gu et al., 2021; La Sorte et al., 2013), and altitudinal (Barve et al., 2016; Ding et al., 2020; Li et al., 2010; Ocampo‐Peñuela & Pimm, 2015). Alternatively, migration can be classified according to how it responds to geographical barriers (e.g., barrier‐crossing migration, detoured migration). It is also reported frequently that some species have a loop‐migration pattern in that they either bypass gigantic geographical barriers (e.g., the Iranian Plateau or the Qinghai‐Tibetan Plateau) from seasonal‐different routes (Ćiković et al., 2021) or only make detours in one season and cross the barriers in another (Mi et al., 2022). A variety of migration strategies have been identified with respect to routes, distances, timing and duration, and the behavior *en* route (Bairlein & Coppack, 2006).

The Qinghai‐Tibetan Plateau, which boasts an average elevation of 4500 m above sea level (Thompson et al., 2000), presents numerous challenges to migratory birds, such as extremely cold weather, low humidity, and declining air density (Altshuler & Dudley, 2006; Butler, 2010). Over time, migratory birds on the plateau have developed unique migration patterns to adapt to this environment (Natarajan et al., 2018; Pan et al., 2017; Parr et al., 2019; Qu et al., 2013). At present, high‐altitude migration studies focuses more on famous migration patterns such as the trans‐Himalayas (Bishop et al., 2015; Li et al., 2020; Literák et al., 2022; Mi et al., 2022), but studies on the migration patterns of endemic migratory birds on the Qinghai‐Tibetan Plateau are scarce.

**FIGURE 1:**
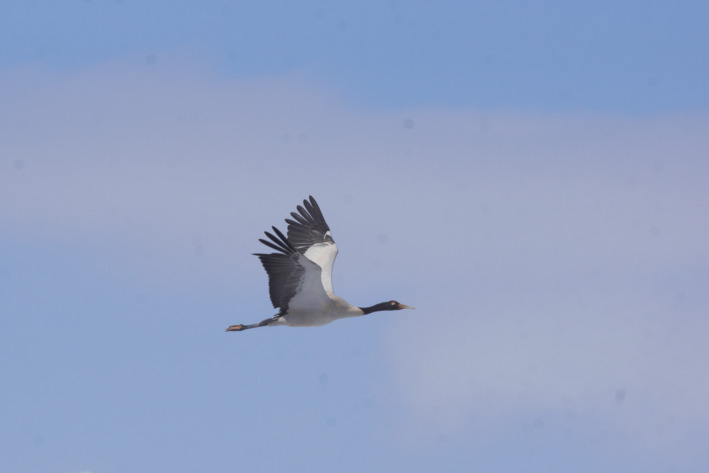
The black‐necked crane (*Grus nigricollis*) is the only alpine crane species. They primarily breed in scattered sites on the Qinghai‐Tibetan Plateau in south‐central China, and winters in southern and eastern parts of the plateau, as well as on the Yunnan‐Guizhou Plateau in China.

The black‐necked crane (*Grus nigricollis*; Figure 1), the only alpine crane species endemic to the plateaus, primarily breeds in scattered sites on the Qinghai‐Tibetan Plateau in south‐central China (Archibald et al., 2020), and winters in southern and eastern parts of the plateau (Bishop et al., 1998; Han & Guo, 2018; Jia et al., 2019), and on the Yunnan‐Guizhou Plateau in China (Archibald et al., 2020; Li, 2014; Zhang et al., 2014). The species has recently been reclassified from “Vulnerable (VU)” to “Near Threatened (NT)” by the International Union for Conservation of Nature (IUCN, 2020) due to recent growth in its population, with an estimated global population of 17,389–17,610 in the wild (Chen et al., 2023). This population growth is attributed to habitat protection and creation measures, as well as a temporary increase in suitable breeding habitats due to glacial melting (Li, 2019). Nevertheless, the species is still facing numerous threats, such as food shortages (Yang & Cangjue, 2013), habitat fragmentation (e.g., power lines and fences; Huang et al., 2023; Wang et al., 2021), habitat destruction (e.g., farming practices, mining activities, and dredging projects; Han & Guo, 2018; Jia et al., 2019), and climate change and anthropogenic pressures (Han et al., 2017, 2018). The black‐necked crane is a crucial environmental indicator and flagship species in the alpine wetland ecosystems due to its unique adaptations. (Li, 2019; Meine & Archibald, 1996). The species is an ideal alpine bird for studying high‐altitude bird migration strategy.

**FIGURE 2:**
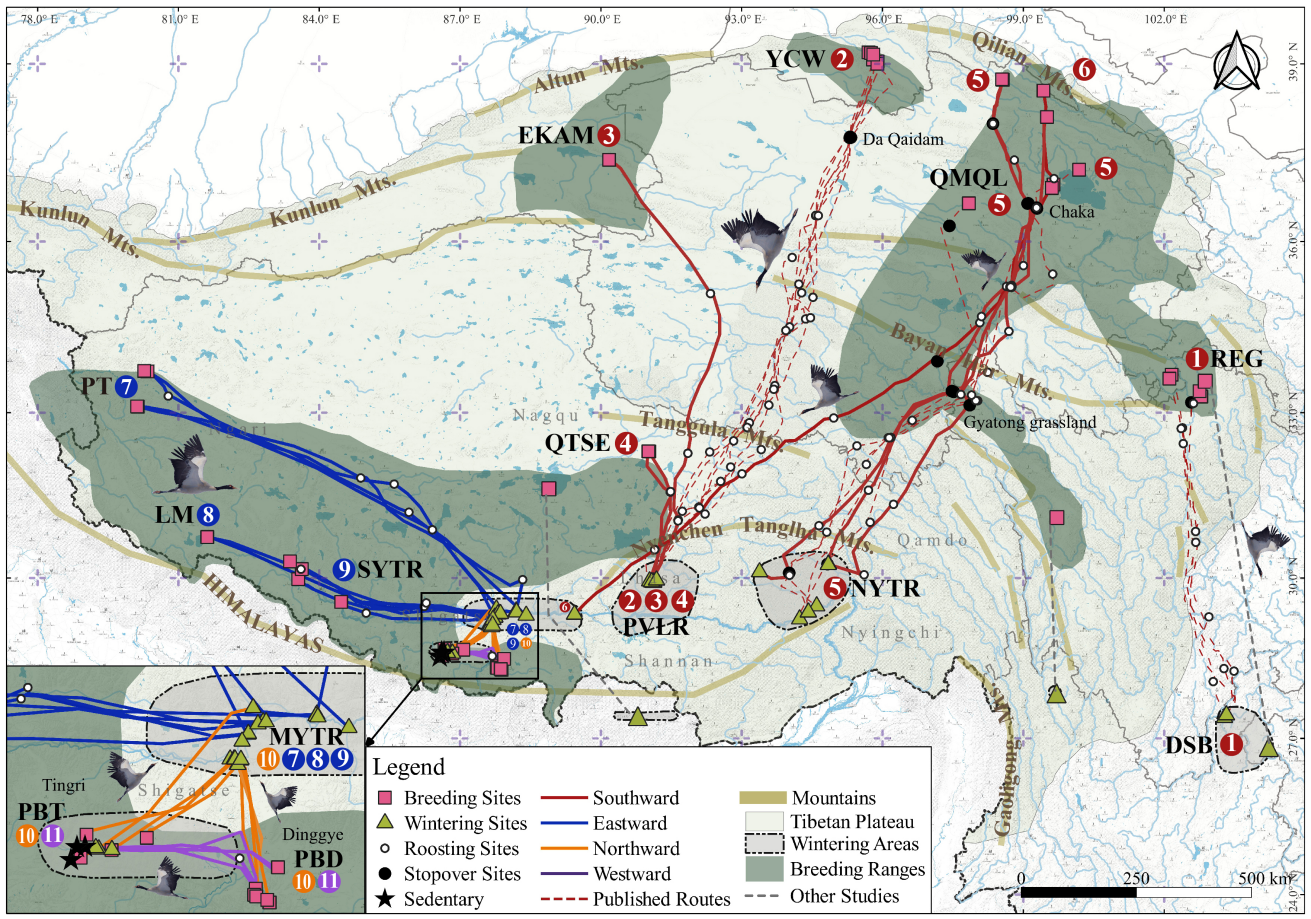
Migration routes of black‐necked cranes. The red dashed line represents published routes in this study, and the gray dashed lines are other tracking studies. The current breeding range was digitized by the author from Li (2014). Detailed information for routes was shown in Tables S1 and S2.

To date, researchers have uncovered five migration routes of black‐necked cranes (Figure 2 red and gray dotted lines): Ruoergai to Dashanbao/Caohai (Qian et al., 2009; Yang et al., 2005), Shenzha to the south slopes of Himalayas (not complete; Archibald, 2005), Shaluli to Napahai (Liu et al., 2012), Qinghai Lake to the estuary of the Ni‐yang and Yarlung Tsangpo Rivers (Wang, Mi, & Guo, 2020), Yanchiwan to Pengbo Valley in the upper reaches of Lhasa River (Wang, Guo, et al., 2020). Despite these valuable contributions, the migration routes connecting several key breeding sites in the hinterland of the Qinghai‐Tibetan Plateau and the northern slopes of the Himalayas have remained undisclosed. To address this knowledge gap, we applied the individual tracking method to explore the autumn migration routes of black‐necked cranes in the aforementioned breeding and wintering areas. Specifically, here we aimed to (1) complete the currently veiled migration routes of black‐necked cranes during autumn migration, (2) identify the spatial–temporal patterns, and (3) compare the altitudinal movement patterns.

## 2 MATERIALS AND METHODS

### 2.1 Field work

From 2015 to 2021, we captured 40 juvenile black‐necked cranes at eight breeding sites using scoop nets (Wang, Guo, et al., 2020) and five adults at three wintering areas using pole traps (Wang, Mi, & Guo, 2020) and fitted them with satellite transmitters. All eight breeding sites located on the Qinghai‐Tibetan Plateau (Figure 2 and Table S1), including Yanchiwan National Nature Reserve (YCW) in Gansu, the east Kunlun and Altun Mountains (EKAM) in Xinjiang, the Qilian Mountains and Qinghai Lake (QMQL) in Qinghai, and Pangong Tso (PT), Lake Manasarovar (LM), the source of the Yarlung Tsangpo River (SYTR), Pumqu Basin in Dinggye (PBD) and Pumqu Basin in Tingri (PBT) in Tibet. The three wintering areas were on the southern Qinghai‐Tibetan Plateau and the northeastern Yunnan‐Guizhou Plateau (Figure 2 and Table S1), including Pengbo Valley in the upper reaches of Lhasa River (PVLR), the estuary of the Ni‐yang and Yarlung Tsangpo River (NYTR) in Tibet, and Dashanbao National Nature Reserve (DSB) in Yunnan. The study area (80°–105° E, 27°–40° N; Figure 2) encompassed nearly all known breeding and wintering sites of black‐necked cranes (Archibald et al., 2020; Li, 2014).

The transmitters (HQBP3622; HQLN0421, HQLG4021S, HQLG4037S, inside diameter 20–22 mm; manufactured by Hunan Global Messenger Technology Co., Ltd) were solar‐charged and weighted 22–44 g. The total mass of each transmitter was less than 2% of the average body mass (over 2 kg) of the cranes, which was below the recommended value in animal telemetry (Barron et al., 2010). We fitted the HQBP3622 transmitter using a back‐pack harness (Thaxter et al., 2014), and the HQLN0421, HQLG4021S, or HQLG4037S transmitter could be attached as a leg‐band on the right leg of each crane (Wang, Guo, et al., 2020). Each crane was released within 10 min after the attachment of satellite tracking transmitters and color rings. The study was approved by the Ethics and Animal Welfare Committee in Beijing Forestry University [EAWC_BJFU], China, with permission from National Forestry and Grassland Administration, China.

### 2.2 Data processing

The juveniles often complete their first autumn migration with their parents (Newton, 2008; Pfister, 1998), and then spend the next 1–2 summers being vagrants until reaching maturity (and consequentially are considered adults; Meine & Archibald, 1996). As a result, the first autumn migration of juveniles is considered to follow the established migration routes of their parents, while their subsequent journeys in the following years were deemed exploratory. Therefore, we recorded the first autumn migration of 40 juvenile cranes in our dataset, excluding their subsequent spring and autumn migrations. To maintain seasonal consistency in the routes, we kept the autumn migration records of five adult cranes, and removed the incomplete tracks due to transmitter failures (e.g., BNC01 and BNC02) and malfunctions (e.g., BNC03 and BNC04), resulting in a collection of 10 complete tracks (each adult with tracks spanning 1–3 years). Thus, this study encompassed a total of 50 tracks (Table S1). We kept only one track from the same family group in the same year for calculations, and thus BNC32, BNC40, and BNC43 were excluded to avoid counting repeatedly. When analyzing five or more tracks, we reported all data as mean ± SD (*N*_track_ ≥ 5); otherwise, we computed only the mean (*N*_track_ < 5).

Data were received hourly via the Global System for Mobile Communications/GSM (CMCC). The tracking data were classified into six accuracy grades: A (±5 m), B (±10 m), C (±20 m), D (±100 m), E (±200 m), and invalid. None of the locations in our dataset fell into the E category. Therefore, we analyzed the data using locations categorized as A, B, C, and D.

### 2.3 Data analysis

We defined cranes as being in migratory flight when the distance between two consecutive locations exceeded 10 km (Mi et al., 2018); cranes that remained in the breeding sites after all other cranes had finished their flights were defined as sedentary (Brown et al., 2021). The autumn migration of black‐necked cranes was defined from the last point recorded in breeding sites (Li, 2014) before departure to the first point recorded in the wintering areas (Chen et al., 2023). Stopover sites referred to sites where cranes stayed for at least 2 days, including long‐term (>10 days) and short‐term (2–10 days) stopover sites, while sites where the cranes stayed for shorter than 2 days were defined as roosting sites (Kölzsch et al., 2015). Furthermore, migration distance was calculated as the sum of all the distances of consecutive migration locations during the migratory flight excluding the variation in locations at the stopover sites (Li et al., 2020). The linear distance was calculated as the distance between the start point and the end point of autumn migration. Migration duration was calculated as the number of days a crane took to complete its autumn migration, including flight duration and stopover duration (Huschle et al., 2013). We calculated flight speed (the daily travel speed = migration distance/flight duration, Nilsson et al., 2013). Step length was calculated as the distance traveled per hour between consecutive migration locations. To address potential errors in step length estimation for short steps due to daytime migration patterns of black‐necked cranes (Wang, Mi, & Guo, 2020), we used the maximum step length observed along each route as a representative measure (Zhao et al., 2017). We calculated the migration distance, linear distance, and maximum step length via the great circle route using the “geosphere” and “move” packages in R v. 4.2.1 (R Core Team, 2022). The migration routes were mapped in QGIS v. 3.24.1 (QGIS Development Team, 2022).

We extracted the ground elevation using latitude and longitude data (Li et al., 2022), calculated the average breeding elevation, average wintering elevation, and the elevation difference between breeding and wintering sites, and compared the ground elevation of different routes in the migratory flight. As the elevation data were not normally distributed (Shapiro–Wilk test, *p* < .05), we applied Wilcoxon rank‐sum test to compare whether the ground elevation of one route was significantly higher than the other. The migration distance finished (%) for each point was calculated as the percentage of its respective migration distance to the overall migration distance. We plotted the ground elevation trends of different routes at the migration distance finished scales, using the smoothing‐fitting method known as locally weighted regression (LOESS) from the “ggplot2” package in R v. 4.2.1 (R Core Team, 2022).

## 3 RESULTS

### 3.1 Migration routes

In our study, we identified 11 autumn migration routes and one sedentary flock, among nine breeding sites and five wintering areas, of which eight routes and the sedentary flock were reported for the first time (Figure 2 and Table 1). Four migration orientations were exhibited among these routes. Six southward flocks primarily bred on the eastern, northern, and central Qinghai‐Tibetan Plateau, with one from the eastern Qinghai‐Tibetan Plateau to the northeastern Yunnan‐Guizhou Plateau, Ruoergai National Nature Reserve (REG) in Sichuan to DSB (along ① REG‐DSB, *n* = 4), and the other five from the northern and central Qinghai‐Tibetan Plateau to south‐central Tibet. Two of them migrated from YCW and EKAM on the northern plateau to PVLR (along ② YCW‐ and ③ EKAM‐PVLR, *n* = 7/2); one from the southeastern Qiangtang (QTSE) in Tibet on the central plateau to PVLR (along ④ QTSE‐PVLR, *n* = 2); two from QMQL on the northeastern plateau to NYTR (⑤ QMQL‐NYTR, *n* = 7) and the middle reaches of the Yarlung Tsangpo River (⑥ QMQL‐MYTR, *n* = 1). The remaining flocks breeding entirely on the northern slopes of the Himalayas showed three eastward flocks and one northward flock migrated to MYTR (along ⑦ PT‐, ⑧ LM‐, ⑨ SYTR‐ and ⑩ PBT/D‐MYTR, *n* = 4/2/4/8), as well as one western flock (along ⑪ PBD‐PBT, *n* = 6) and three sedentary individuals overwintered in PBT (⑫; black stars in Figure 2).

**TABLE 1:** Migration parameters of 11 migration routes and one sedentary flock.

Routes		Breeding sites	Wintering areas	*N*_track_	Departure date	Arrival date	Duration (days)		Distance (km)		Flight speed (km/day)	maximum Step length (km)
							Migration	Flight	Migration	Linear		
**S**	①	**REG**	**DSB**	4	Nov 6–12	Nov 12–16	5.25	3.75	773.26	677.34	208.09	82.10
	②	**YCW**	**PVLR**	7	Sep 29 to Oct 28	Nov 7–18	32.71 ± 10.42	6 ± 1.53	1192.47 ± 42.45	1107.14 ± 5.3	210.37 ± 54.23	67.21
	③	**EKAM**		1 (2)	Oct 31	Nov 3	4	4	954.08	830.02	238.52	78.53
	④	**QTSE**		2	Oct 27	Oct 28	2	2	294.68	262.87	147.34	60.78
	⑤	**QMQL**	**NYTR**	7	Sep 26 to Oct 31	Nov 9–20	31 ± 11.25	7 ± 1	1176.92 ± 76.33	984.11 ± 53.25	169.69 ± 13.52	62.00
	⑥		**MYTR**	1	Oct 20	Nov 1	13	9	1519.06	1377.08	168.78	66.28
**E**	⑦	**PT**	**MYTR**	4	Oct 11 to Nov 4	Oct 13 to Nov 5	2.5	2.5	923.45	880.11	384.43	107.78
	⑧	**LM**		2	Oct 31/Nov 4	Nov 1/Nov 5	2	2	654.33	637.33	327.17	100.36
	⑨	**SYTR**		4	Oct 17–26	Oct 17–27	1.5	1.5	418.19	397.87	319.83	92.07
**N**	⑩	**PBT/D**		8	Nov 14–23	Nov 14–23	1 ± 0	1 ± 0	100.62 ± 22.32	97.59 ± 22.47	100.62 ± 22.32	83.65
**W**	⑪	**PBD**	**PBT**	4 (6)	Oct 30 to Nov 23	Oct 30 to Nov 23	1.25	1.25	124.63	111.49	108.46	54.36
⑫ **Sedentary (PBT)**				3	‐	‐	‐	‐	‐	5.05	‐	‐

*Note*: All data are presented as mean ± SD (*N*_track_ ≥ 5) or the mean (*N*_track_ < 5). BNC40 (③), as well as BNC32 and BNC43 (⑪) were excluded to avoid counting repeatedly.

We found that the breeding colonies, which were adjacent to the northern slopes of the Himalayas, migrated to wintering areas (e.g., MYTR and PBT) in different orientations (Figure 2). In addition, we recorded two breeding colonies migrating to different wintering areas via alternative routes, such as eight individuals along ⑤ QMQL‐NYTR (*n* = 7) and ⑥ QMQL‐MYTR (*n* = 1), 10 individuals along ⑩ PBD‐MYTR (*n* = 4) and ⑪ PBD‐PBT (*n* = 6).

### 3.2 Spatial–temporal patterns

The flocks following different routes showed different spatial–temporal patterns in terms of migration distances, duration (migration and stopover), flight speed, and maximum step lengths (Figure 2 and Table 1). Three southward flocks bred in the Qilian Mountains, with migration distances exceeding 1000 km, including ② YCW‐PVLR, ⑤ QMQL‐NYTR, and ⑥ QMQL‐MYTR, among which ⑥ was the longest (1519.06 km; Table S2). The three southward flocks departed earlier than the average departure date (Oct 28) and had the longest migration durations due to stopovers, but they were not the slowest. As shown in Figure 2 and Table 2, the flocks along ② and ⑤ stayed in the long‐term stopover sites, located in the hypersaline lakes (Da Qaidam and Chaka), for 26.71 ± 10.00 days (*n* = 7) and 25.40 ± 9.32 days (*n* = 5), respectively. Some cranes (along ⑤ and ⑥) migrated to Gyatong grassland for a short‐term stopover (5 days, *n* = 3; 4 days, *n* = 1), followed by different wintering areas. The other three flocks (along ① REG‐DSB, ③ EKAM‐ and ④ QTSE‐PVLR) bred on eastern, northern, and central Qinghai‐Tibetan Plateau, excluding one individual along ① using the stopover site (Table 2), migrated for 2–5 days (Table S2). Three eastward flocks that bred on the northern slopes of the Himalayas (along ⑦ PT‐, ⑧ LM‐, and ⑨ SYTR‐MYTR) showed the most urgent patterns during autumn migration (Table 1). Specifically, they had flight speeds exceeding 300 km/days (with ⑦ being the fastest) and the longest maximum step lengths. Two flocks, one northward and one westward (along ⑩ PBT/D‐MYTR and ⑪ PBD‐PBT), bred in the Pumqu Basin on the northern slopes of the Himalayas, with migration distance less than 200 km, and ⑩ was the shortest (100.62 ± 22.32 km; Table 1). Both flocks departed later than the average and spent the shortest migration durations (1–2 days) due to the short distance. In addition, we observed that both flocks had the slowest flight speed, but the northward flock had longer maximum step lengths than the westward flock.

**TABLE 2:** Stopover sites of black‐necked cranes' autumn migration routes from breeding sites to wintering areas.

Routes	Breeding sites	ID	Departure date	Duration of arrival and departure at each stopover sites (days)														Arrival date	Wintering areas
				**Sichuan (SC)**															
				Hongyuan					Baoxing										
①	**REG**	BNC 02	Nov 6, 2016	**4**					+									Nov 12, 2016	**DSB**
				**Qinghai**										**Tibet**					
				Da Qaidam	Golmud		Qumarleb		Zhidoi			Zadoi		Nagqu		Lhasa			
②	**YCW**	BNC 05	Oct 25, 2018	**16**	+				+			+		+/+				Nov 16, 2018	**PVLR**
		BNC 06	Oct 7, 2018	**30**			+/+					+		+				Nov 11, 2018	
		BNC 07	Oct 28, 2019	**15**			+/+					+		+/+				Nov 18, 2019	
		BNC 08	Sep 29, 2019	**42**	+		+					+		+/+		+		Nov 17, 2019	
		BNC 11	Oct 3, 2020	**36**	+				+					+/+				Nov 13, 2020	
		BNC 12	Oct 12, 2020	**25**	+									+				Nov 9, 2020	
		BNC 13	Oct 12, 2020	**23**					+					+				Nov 7, 2020	
				**Qinghai**							**SC**	**Qinghai**		**Tibet**					
				Tianjun	Gonghe	Chaka	Dulan	Xinghai	Madoi	Chindu	Shiqu	Yushu	Nanqian	Qamdo	Nyingchi	Nagqu	Lhasa		
⑤	**QMQL**	BNC 03	Oct 31, 2016				**8**				+	+		+	**7**			Nov 20, 2016	**NYTR**
		BNC 04	Sep 26, 2016			**39**	+				+		+/+					Nov 9, 2016	
			Oct 12, 2017			**25**			+		+		+/+		+			Nov 13, 2017	
			Oct 24, 2018			**13**		+	+		+		+	+				Nov 12, 2018	
		BNC 10	Oct 4, 2020	**2**/+		**23**			+	**6**				+				Nov 10, 2020	
		BNC 25	Oct 4, 2021	+		**27**			+/+	+	**4**			+	+			Nov 14, 2021	
		BNC 26	Oct 23, 2021			+			+/+	**5**				+	**6**			Nov 9, 2021	
⑥		BNC 09	Oct 20, 2020		+	+			+/+	**4**			+			+	+	Nov 1, 2020	**MYTR**

*Note*: “+” and “+/+” represent 1 roosting site and 2 roosting sites respectively.

### 3.3 Altitudinal movement patterns

**FIGURE 3:**
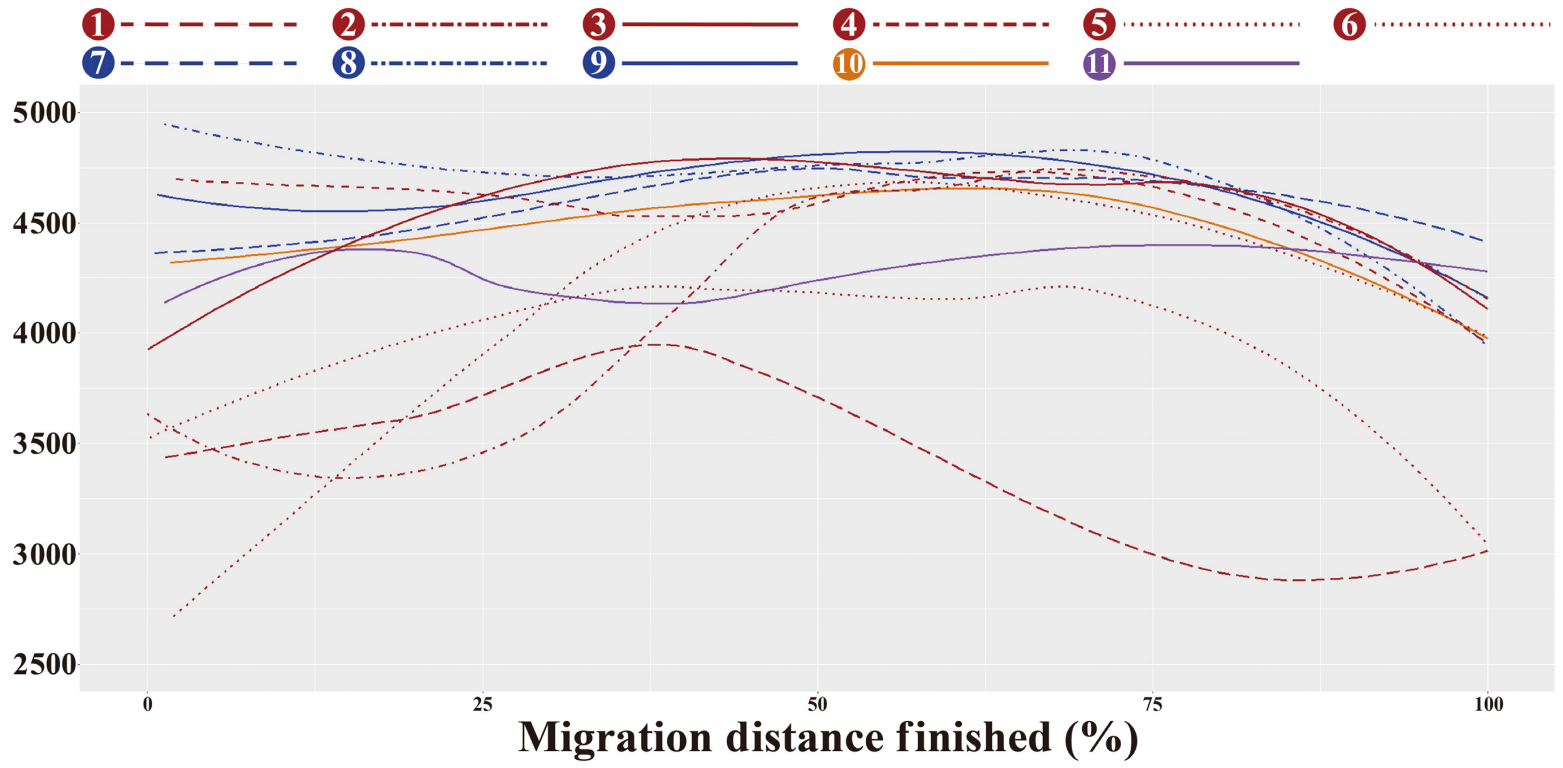
The ground elevation trends of different routes at the migration distance finished scales. The ground elevation trends of both flocks along ⑤ and ⑥, represented by the same color and linetype on the plot, indicated that the two flocks migrated from the same breeding site (the Qilian Mountains and Qinghai Lake in Qinghai).

Different flocks showed distinct altitudinal movement patterns during autumn migration with breeding elevations, elevation differences, ground elevations, and elevation trends, especially for southward flocks. One southward flock migrated from the eastern Qinghai‐Tibetan Plateau to the northeastern Yunnan‐Guizhou Plateau (along ① REG‐DSB), following the lowest route (*p* < .0001; Table 3 and Figure 3). Three southward flocks bred in the Qilian Mountains on the northeastern Qinghai‐Tibetan Plateau (along ② YCW‐PVLR, ⑤ QMQL‐NYTR, and ⑥ QMQL‐MYTR), and their breeding elevations were lower than other flocks (Table 3). During the flight, the ground elevation along ② was significantly higher than ⑤ and ⑥ (*p* < .0001; Table 3), as well as ⑤ was significantly below ⑥ (*p* < .0001; Table 3). All ② and most ⑤ selected the long‐term stopover sites at ground elevations close to their breeding sites (Da Qaidam: 3157.14 ± 9.73 m and Chaka: 3074.22 ± 11.37 m). The three southward flocks faced significant altitude ascent and prolonged flights at high altitudes (Figure 3). After flying over the final east–west mountains range (Nyenchen Tanglha Mts. In Figure 2), the flock along ⑤ migrated to NYTR with lower elevation (elevation difference: −499.57 m; Table 3), while ② to PVLR and ⑥ to MYTR with higher elevation (elevation difference: 708.14 m and 446.00 m; Table 3). Two other southward flocks bred on the northern and central Qinghai‐Tibetan Plateau (along ③ EKAM‐ and ④ QTSE‐PVLR), had significantly higher ground elevations (*p* < .05; Table 3) than the above southward flocks. The flock along ④ had higher breeding sites than the flock along ③, but significantly lower ground elevations (*p* < .0001; Table 3). The latter flock (along ③) faced a gentle and prolonged flight (Figure 3) and showed no significant elevation difference (Table 3). Three eastward flocks bred on the northern slopes of the Himalayas (along ⑦ PT‐, ⑧ LM‐, and ⑨ SYTR‐MYTR), migrated significantly higher ground elevations than other flocks (excluding ③; *p* < .05; Table 3), and flew to the same wintering area with lower altitude (Table 3). Two flocks, one northward and one westward (along ⑩ PBT/D‐MYTR and ⑪ PBD‐PBT), bred in the Pumqu Basin on the northern slopes of the Himalayas. The northward flock experienced a short altitude ascent (~150 km; Table S1 and Figure 3), while the westward flock showed a gentle elevation change (Figure 3).

**TABLE 3:** Ground elevation of the autumn migration routes of black‐necked cranes.

Routes		Breeding sites	Breeding elevation (m)	Wintering areas	Elevation difference (m)	Flight elevation (m)	Wilcoxon rank‐sum test										
							①	②	③	④	⑤	⑥	⑦	⑧	⑨	⑩	⑪
**S**	①	**REG**	3446.00	**DSB**	−357.25	3231.42 ± 688.51											
	②	**YCW**	3178.14	**PVLR**	708.14	4311.27 ± 653.34	****				****	****				**	****
	③	**EKAM**	3891.00		−37.00	4640.45 ± 260.64	****	****		****	****	****			ns	****	****
	④	**QTSE**	4639.00		−728.50	4572.69 ± 210.03	****	*			****	****				****	****
	⑤	**QMQL**	3338.00	**NYTR**	−499.57	3997.82 ± 520.86	****										
	⑥			**MYTR**	446.00	4094.68 ± 611.79	****				****					ns	ns
**E**	⑦	**PT**	4398.25	**MYTR**	−482.00	4700.46 ± 331.5	****	****	*	***	****	****		ns	ns	****	****
	⑧	**LM**	4593.00		−667.50	4717.09 ± 265.57	****	****	****	****	****	****	ns		*	****	****
	⑨	**SYTR**	4612.50		−659.00	4656.84 ± 269.31	****	****	ns	**	****	****	ns			****	****
**N**	⑩	**PBT/D**	4273.75		−285.50	4356.92 ± 365.68	****				****	ns					**
**W**	⑪	**PBD**	4203.75	**PBT**	104.25	4251.22 ± 184.16	****				****	ns					

Abbreviation: ns, no significance.*p < .05; **p < .01; ***p < .001; ****p < .0001. The significance shows whether the ground elevation of this route was significantly higher than others.

## 4 DISCUSSION

We identified 11 autumn migration routes and one sedentary flock, connecting nine breeding sites and five wintering areas for black‐necked cranes, with eight routes and the sedentary flock being reported for the first time. Four distinct migration orientations were observed among these routes. We found considerable variations in the spatial–temporal patterns of flocks following different routes in terms of migration distances, duration (migration and stopover), flight speed, and maximum step lengths. Furthermore, there were significant disparities in the altitudinal movement patterns of different flocks with breeding elevations, elevation differences, ground elevations, and elevation trends.

Spatially, crane species in East Asia typically engage in long‐distance migration from higher to lower latitudes for overwintering, often covering continental‐scale distance. For example, Siberian Crane (*Leucogeranus leucogeranus* 5586 km; Kanai et al., 2002), Hooded Crane (*G. monacha* 3800 km; Higuchi et al., 1992), and White‐naped Crane (*Antigone vipio* 2558 km; Higuchi et al., 2004). Occasionally, some species, such as the Red‐crowned Cranes (*G. japonensis*), can be sedentary in Hokkaido (Masatomi & Masatomi, 2018). Previous studies on the migration routes of black‐necked cranes have primarily focused on the Yunnan‐Guizhou Plateau or the eastern and northeastern Qinghai‐Tibetan Plateau, revealing the southward routes to the lower latitude wintering areas during autumn migration (see the gray and red dashed lines in Figure 2; Archibald, 2005; Liu et al., 2012; Qian et al., 2009; Wang, Guo, et al., 2020; Wang, Mi, & Guo, 2020; Yang et al., 2005). In our study, we found that black‐necked cranes migrated at a smaller scale (~1520 km) compared to those crane species, perhaps due to their limited suitable habitat in central Asian uplands. Southward and eastward flocks also migrated to the lower latitude wintering areas (e.g., DSB in Yunnan, NYTR, PVLR, and MYTR in Tibet). Interestingly, two eastward flocks demonstrated this by predominantly longitudinal rather than latitudinal shift (⑧ LM‐ and ⑨ SYTR‐MYTR; Figure 2). In addition, the flocks breeding in the Pumqu Basin on the northern slopes of the Himalayas displayed northward migration(⑩ PBT/D‐MYTR) and westward migration (⑪ PBD‐PBT), or sedentary behavior (⑫; black stars in Figure 2).

Temporally, cranes bred at higher latitudes departed earlier than those bred at lower latitudes, possibly due to deteriorating conditions (e.g., cold weather and food scarcity; Halupka et al., 2017; Newton, 2008). As shown in Figure 2 and Table 2, the YCW flock selected Da Qaidam as a long‐term stopover site (26.71 ± 10.00 days, *n* = 7), while the QMQL flock exhibited more dispersed in stopover sites and durations, with long‐term stopover mainly in Chaka (25.40 ± 9.32 days, *n* = 5) and short‐term in Gyatong grassland (5 days, *n* = 3; 4 days, *n* = 1). In addition, some flocks (along ② YCW‐PVLR and ⑤ QMQL‐NYTR) selected hypersaline lakes (Da Qaidam and Chaka) as their long‐term stopover sites, indicating that highly saline habitats may provide unique habitats and food resources (Bairlein, 2003; Muzaffar et al., 2008).

The elevation was tested as the most important environmental variable in the breeding habitat selection of black‐necked cranes (Han et al., 2018), and our results also revealed that the breeding colonies (YCW and QMQL) in the Qilian Mountains on the northeastern Qinghai‐Tibetan Plateau used long‐term stopover sites before embarking on significant altitude ascent (e.g., ② YCW‐PVLR and ⑤ QMQL‐NYTR; Figure 3), while other flocks displayed more urgent migration patterns, preferring to roost only at night. Even though these two flocks exhibited different stopover patterns on the horizontal, our results showed that they almost all selected the long‐term stopover sites at a ground elevation close to their breeding sites (Da Qaidam: 3157.14 ± 9.73 m and Chaka: 3074.22 ± 11.37 m). These demonstrate that the decision to stopover and the duration in stopover sites may be associated with food availability and weather conditions at the stopover sites (Alonso et al., 1990a, 1990b, 1994; Swanberg, 1987; Volkov et al., 2016), as well as refueling needs for the subsequent altitude ascent and prolonged flights.

The remarkable diversity in the migration routes of black‐necked cranes, especially in terms of migration orientations, spatial–temporal patterns, and altitudinal movement patterns, could be attributed to three main factors. First, these routes were determined by various geography and topography. The significant altitude ascent perhaps influenced the stopover decision for the breeding colonies in the Qilian Mountains (along ② YCW‐PVLR and ⑤ QMQL‐NYTR; Figures 2 and 3). Moreover, the Himalayas as a geographical barrier, might force some flocks to detour along river valleys in alternative orientations (along ⑧ LM‐ and ⑨ SYTR‐MYTR; Figure 2; Hahn et al., 2014), or follow other routes offering wetland areas for feeding and resting (along ⑦ PT‐MYTR; Figure 2; Wang, Mi, & Guo, 2020). However, the Himalayas did not deter all black‐necked cranes, as previous studies have recorded an alternate wintering strategy of crossing the Himalayas and overwintering on the southern slopes (Mahar et al., 2022). Due to the high flight costs and limited habitat availability (Hawkes et al., 2011), only a small number of cranes (about 500 individuals) overwinter on the southern slopes each year (Phuntsho & Tshering, 2014). Notably, the sedentary flock in the PBT was reported for the first time, which may be associated with the abundant local geothermal resources (Johnsgard, 1983).

Secondly, black‐necked cranes possessed distinctive ecological habits and physiological properties. The species demonstrated dispersed breeding and concentrated wintering behaviors (Archibald et al., 2020), such that the breeding colonies were scattered around their wintering areas, resulting in diverse migration orientations towards their wintering area (e.g., MYTR). Additionally, black‐necked cranes, being highly dependent on highland wetland ecosystems, could better tolerate the extreme wintering conditions in the highlands compared to other migratory birds crossing the Himalayas (Bishop et al., 2015; Li et al., 2020; Literák et al., 2022; Mi et al., 2022).

Finally, the breeding colonies in the Pumqu Basin (e.g., PBT/D) might tend to be categorized as dispersive migration (outward‐return movement in various directions) rather than migration (regular return movement in fixed directions). The PBT/D flocks displayed relatively shorter (up to 200 km; Table S2) and more random movements (northward, westward, or sedentary), not related to seasonal changes in food supplies and significant changes in the species' latitudinal distribution or altitudinal center (Figure 2 and Table 3; Newton, 2008), unlike the southward and eastward flocks. However, further research is needed to verify the migration category of the breeding colonies.

Our results revealed multiple routes that connect different geographical breeding colonies to the same wintering areas, while the northern slopes of the Himalayas (e.g., MYTR, PBT, PVLR, and NYTR in Tibet; Figure 2) might be more suitable wintering areas for black‐necked cranes than the southern slopes. Although these breeding and wintering areas are all located within established nature reserves (Chen et al., 2023), the species is still facing various threats (Han et al., 2017; Han & Guo, 2018; Jia et al., 2019; Wang et al., 2021; Yang & Cangjue, 2013). Regarding the two long‐term stopover sites (Da Qaidam and Chaka), the species' stopover duration also coincided with the tourist season for hypersaline lakes. To address this issue, we propose implementing regular patrols (at least 36 days) at these important sites during autumn migration and increasing education efforts to raise animal protection awareness among visitors (Wang, Guo, et al., 2020; Wang, Mi, & Guo, 2020). Our results also highlighted the roosting sites and short‐term stopover sites likely constituted an important component of the migration routes. Therefore, it is critical to discover their critical habitats and connectivity among them, further to develop effective seasonal conservation plans.

We considered that juveniles could be representative of their breeding colonies, as juveniles followed the established migration routes of their parents (breeding pairs) during their first autumn migration. However, black‐necked cranes, being a long‐lived migrant highly dependent on plateaus, might show their behavior could vary according to different factors, such as age, season, weather, or habitat conditions (Abrahms et al., 2021; Mi et al., 2022; Newton, 2004). Furthermore, due to the limited sample size, particularly the flock along ⑥ QMQL‐MYTR (*n* = 1) without field verification, uncertainty remained regarding whether this tracked crane was a vagrant or followed an alternative route. Consequently, further research on black‐necked crane migration is essential to better understand their migration patterns and strategies.

Despite the limitations, our study revealed the remarkable diversity in the migration routes of black‐necked cranes, particularly in terms of migration orientations, spatial–temporal patterns, and altitudinal movement patterns. Four distinct migration orientations were observed among these routes. Furthermore, we expanded the known range of migration distances to 84–1520 km at both ends (excluding sedentary individuals) and identified two long‐term stopover sites (Da Qaidam and Chaka) and one short‐term stopover site (Gyatong grassland). Additionally, we found that the breeding colonies (YCW and QMQL) in the Qilian Mountains on the northeastern Qinghai‐Tibetan Plateau used long‐term stopover sites before embarking on significant altitude ascent, while other flocks displayed more urgent migration patterns, preferring to roost only at night. In conclusion, our study provides valuable insights into the near‐complete migration routes of black‐necked cranes. Further research on the migration routes of black‐necked cranes will help to uncover the spatial–temporal behaviors and migration strategies of migratory birds inhabiting high‐altitude areas.

## AUTHOR CONTRIBUTIONS

**Zhen Pu:** Data curation (lead); formal analysis (lead); funding acquisition (supporting); investigation (equal); visualization (lead); writing – original draft (lead); writing – review and editing (equal). **Yumin Guo:** Conceptualization (lead); data curation (supporting); funding acquisition (lead); investigation (equal); supervision (lead); writing – review and editing (equal).

## CONFLICT OF INTEREST STATEMENT

The authors declare that they have no competing interests.

## Supporting information


Tables S1–S2.

## Data Availability

The data that support the findings of this study are openly available in Dryad at https://doi.org/10.5061/dryad.47d7wm3jk.
