# Supplementary material for: Autumn migration of black‐necked crane (Grus nigricollis) on the Qinghai‐Tibetan and Yunnan‐Guizhou plateaus
Source: Ecol Evol. 2023 Sep 7;13(9):e10492. doi: 10.1002/ece3.10492 (PMC10485337; doi:10.1002/ece3.10492)
Supplement: Supplementary file 1 — Tables S1–S2. [file ECE3-13-e10492-s001.docx]

**Table S1 The detail information of breeding sites, wintering areas, and 45 tracked cranes.**

| **Routes** | | **Breeding**  **Sites** | **Wintering**  **Areas** | **ID** | **Adult/**  **Juvenule** | **Family**  **Group** | **N*_group_*** | **Year** | | | | | | | **N*_track_*** |
| --- | --- | --- | --- | --- | --- | --- | --- | --- | --- | --- | --- | --- | --- | --- | --- |
|  |  |  |  |  |  |  |  | **2015** | **2016** | **2017** | **2018** | **2019** | **2020** | **2021** |  |
| **S** | ① | **REG**  (Ruoergai NNR, Sichuan) | **DSB** (Dashanbao NNR, Yunnan) | BNC01 | A | I | 2 | **√** | **√** |  |  |  |  |  | 4 |
|  |  |  |  | BNC02 | A | II |  | **√** | **√** |  |  |  |  |  |  |
|  | ② | **YCW**  (Yanchiwan NNR, Gansu) | **PVLR** (Pengbo Valley in the upper reaches of Lhasa River, Tibet) | BNC05 | J | I | 7 |  |  |  | √ |  |  |  | 7 |
|  |  |  |  | BNC06 | J | II |  |  |  |  | √ |  |  |  |  |
|  |  |  |  | BNC07 | J | III |  |  |  |  |  | √ |  |  |  |
|  |  |  |  | BNC08 | J | IV |  |  |  |  |  | √ |  |  |  |
|  |  |  |  | BNC11 | J | V |  |  |  |  |  |  | √ |  |  |
|  |  |  |  | BNC12 | J | VI |  |  |  |  |  |  | √ |  |  |
|  |  |  |  | BNC13 | J | VII |  |  |  |  |  |  | √ |  |  |
|  | ③ | **EKAM**  (The east Kunlun and Altun Mountains, Xinjiang) |  | BNC39 | J | I | 1 |  |  |  |  |  |  | √ | 2 |
|  |  |  |  | BNC40 | J | I |  |  |  |  |  |  |  | √ |  |
|  | ④ | **QTSE**  (The southeastern Qiangtang, Tibet) |  | BNC14 | A | I | 1 |  |  |  |  |  | **√** | **√** | 2 |
|  | ⑤ | **QMQL**  (The Qilian Mountains and Qinghai Lake, Qinghai) | **NYTR**  (The estuary of the Ni-yang and Yarlung Tsangpo River, Tibet) | BNC03 | A | I | 6 |  | **√** |  |  |  |  |  | 7 |
|  |  |  |  | BNC04 | A | II |  |  | **√** | **√** | **√** |  |  |  |  |
|  |  |  |  | BNC10 | J | III |  |  |  |  |  |  | √ |  |  |
|  |  |  |  | BNC25 | J | IV |  |  |  |  |  |  |  | √ |  |
|  |  |  |  | BNC26 | J | V |  |  |  |  |  |  |  | √ |  |
|  | ⑥ |  | **MYTR** | BNC09 | J | VI |  |  |  |  |  |  | √ |  | 1 |
| **E** | ⑦ | **PT**  (Pangong Tso, Tibet) | **MYTR** | BNC35 | J | I | 4 |  |  |  |  |  |  | √ | 4 |
|  |  |  |  | BNC36 | J | II |  |  |  |  |  |  |  | √ |  |
|  |  |  |  | BNC37 | J | III |  |  |  |  |  |  |  | √ |  |
|  |  |  |  | BNC38 | J | IV |  |  |  |  |  |  |  | √ |  |
|  | ⑧ | **LM**  (Lake Manasarovar, Tibet) |  | BNC33 | J | I | 2 |  |  |  |  |  |  | √ | 2 |
|  |  |  |  | BNC34 | J | II |  |  |  |  |  |  |  | √ |  |
|  | ⑨ | **SYTR** |  | BNC21 | J | I | 4 |  |  |  |  |  | √ |  | 4 |
|  |  |  |  | BNC22 | J | II |  |  |  |  |  |  | √ |  |  |
|  |  | (The source of the Yarlung Tsangpo River, Tibet) | (The middle reaches of the Yarlung Tsangpo River, Tibet) | BNC23 | J | III |  |  |  |  |  |  | √ |  |  |
|  |  |  |  | BNC24 | J | V |  |  |  |  |  |  | √ |  |  |
| **N** | ⑩ | **PBT/D** |  | BNC15 | J | I | 8 |  |  |  |  |  | √ |  | 8 |
|  |  |  |  | BNC16 | J | II |  |  |  |  |  |  | √ |  |  |
|  |  |  |  | BNC20 | J | III |  |  |  |  |  |  | √ |  |  |
|  |  |  |  | BNC41 | J | IV |  |  |  |  |  |  | √ |  |  |
|  |  |  |  | BNC42 | J | V |  |  |  |  |  |  | √ |  |  |
|  |  |  |  | BNC28 | J | VI |  |  |  |  |  |  |  | √ |  |
|  |  |  |  | BNC29 | J | VII |  |  |  |  |  |  |  | √ |  |
|  |  |  |  | BNC30 | J | VIII |  |  |  |  |  |  |  | √ |  |
| **W** | ⑪ | **PBD**  (Pumqu Basin in Dinggye, Tibet) | **PBT**  (Pumqu Basin in Tingri, Tibet) | BNC18 | J | I | 4 |  |  |  |  |  | √ |  | 6 |
|  |  |  |  | BNC43 | J | I |  |  |  |  |  |  | √ |  |  |
|  |  |  |  | BNC19 | J | II |  |  |  |  |  |  | √ |  |  |
|  |  |  |  | BNC44 | J | III |  |  |  |  |  |  | √ |  |  |
|  |  |  |  | BNC31 | J | IV |  |  |  |  |  |  |  | √ |  |
|  |  |  |  | BNC32 | J | IV |  |  |  |  |  |  |  | √ |  |
| ⑫ **Sedentary (PBT)** | | | | BNC17 | J | I | 3 |  |  |  |  |  | √ |  | 3 |
|  |  |  |  | BNC45 | J | II |  |  |  |  |  |  | √ |  |  |
|  |  |  |  | BNC27 | J | III |  |  |  |  |  |  |  | √ |  |
| **Sum** | | | | **45** |  |  | **42** | **2** | **4** | **1** | **3** | **2** | **21** | **17** | **50** |

**Table S2 Autumn migration parameters of black-necked cranes in 2015-2021.**

| **Routes** | | **ID** | **Year** | **Departure**  **Date** | **Arrival**  **Date** | **Duration (d)** | | | **N_Roosting_** | **Distance (km)** | | **Flight**  **Speed (km/d)** |
| --- | --- | --- | --- | --- | --- | --- | --- | --- | --- | --- | --- | --- |
|  |  |  |  |  |  | Migration | Flight | Stopover |  | Migration | Linear |  |
| **S** | ① | BNC01 | 2015 | Nov 12 | Nov 15 | 4 | 4 |  | 3 | 840.69 | 700.24 | 210.17 |
|  |  |  | 2016 | Nov 12 | Nov 16 | 5 | 4 |  | 3 | 741.71 | 692.95 | 185.43 |
|  |  | BNC02 | 2015 | Nov 10 | Nov 14 | 5 | 4 |  | 3 | 807.60 | 655.20 | 201.90 |
|  |  |  | 2016 | Nov 6 | Nov12 | 7 | 3 | 4 | 1 | 703.05 | 660.96 | 234.35 |
|  | ② | BNC05 | 2018 | Oct 25 | Nov 16 | 23 | 7 | 16 | 5 | 1190.41 | 1111.40 | 170.06 |
|  |  | BNC06 |  | Oct 7 | Nov 11 | 36 | 6 | 30 | 4 | 1194.69 | 1102.70 | 199.11 |
|  |  | BNC07 | 2019 | Oct 28 | Nov 18 | 22 | 7 | 15 | 5 | 1262.64 | 1102.78 | 180.38 |
|  |  | BNC08 |  | Sep 29 | Nov 17 | 50 | 8 | 42 | 6 | 1229.16 | 1111.50 | 153.64 |
|  |  | BNC11 | 2020 | Oct 3 | Nov 13 | 42 | 6 | 36 | 4 | 1178.58 | 1110.84 | 196.43 |
|  |  | BNC12 |  | Oct 12 | Nov 9 | 29 | 4 | 25 | 2 | 1147.10 | 1099.35 | 286.77 |
|  |  | BNC13 |  | Oct 12 | Nov 7 | 27 | 4 | 23 | 2 | 1144.70 | 1111.38 | 286.17 |
|  | ③ | BNC39 | 2021 | Oct 31 | Nov 3 | 4 | 4 |  | 3 | 954.08 | 830.02 | 238.52 |
|  |  | BNC40 |  | Oct 31 | Nov 3 | 4 | 4 |  | 3 | 956.88 | 830.10 | 239.22 |
|  | ④ | BNC14 | 2020 | Oct 27 | Oct 28 | 2 | 2 |  | 1 | 283.13 | 261.84 | 141.57 |
|  |  |  | 2021 | Oct 27 | Oct 28 | 2 | 2 |  | 1 | 306.22 | 263.89 | 153.11 |
|  | ⑤ | BNC03 | 2016 | Oct 31 | Nov 20 | 21 | 6 | 8/7 | 3 | 1081.40 | 888.76 | 180.23 |
|  |  | BNC04 | 2016 | Sep 26 | Nov 9 | 45 | 6 | 39 | 4 | 1105.97 | 954.82 | 184.33 |
|  |  |  | 2017 | Oct 12 | Nov 13 | 33 | 7 | 25 | 5 | 1216.94 | 1023.74 | 173.85 |
|  |  |  | 2018 | Oct 24 | Nov 12 | 20 | 7 | 13 | 5 | 1201.11 | 966.78 | 171.59 |
|  |  | BNC10 | 2020 | Oct 4 | Nov 10 | 38 | 7 | 2/23/6 | 3 | 1123.22 | 1000.65 | 160.46 |
|  |  | BNC25 | 2021 | Oct 4 | Nov 14 | 42 | 9 | 27/4 | 6 | 1297.15 | 1002.10 | 144.13 |
|  |  | BNC26 |  | Oct 23 | Nov 9 | 18 | 7 | 5/6 | 4 | 1212.65 | 1051.92 | 173.24 |
|  | ⑥ | BNC09 | 2020 | Oct 20 | Nov 1 | 13 | 9 | 4 | 7 | 1519.06 | 1377.08 | 168.78 |
| **E** | ⑦ | BNC35 | 2021 | Oct 11 | Oct 13 | 3 | 3 |  | 2 | 956.51 | 871.54 | 318.84 |
|  |  | BNC36 |  | Oct 12 | Oct 13 | 2 | 2 |  | 1 | 914.33 | 874.45 | 457.17 |
|  |  | BNC37 |  | Oct 26 | Oct 28 | 3 | 3 |  | 2 | 898.57 | 858.80 | 299.52 |
|  |  | BNC38 |  | Nov 4 | Nov 5 | 2 | 2 |  | 1 | 924.38 | 915.66 | 462.19 |
|  | ⑧ | BNC33 | 2021 | Oct 31 | Nov 1 | 2 | 2 |  | 1 | 666.36 | 652.37 | 333.18 |
|  |  | BNC34 |  | Nov 4 | Nov 5 | 2 | 2 |  | 1 | 642.30 | 622.29 | 321.15 |
|  | ⑨ | BNC21 | 2020 | Oct 26 | Oct 27 | 2 | 2 |  | 1 | 328.42 | 319.14 | 164.21 |
|  |  | BNC22 |  | Oct 24 | Oct 25 | 2 | 2 |  | 1 | 458.47 | 413.26 | 229.24 |
|  |  | BNC23 |  | Oct 26 | Oct 26 | 1 | 1 |  |  | 429.17 | 417.52 | 429.17 |
|  |  | BNC24 |  | Oct 17 | Oct 17 | 1 | 1 |  |  | 456.70 | 441.54 | 456.70 |
| **N** | ⑩ | BNC15 | 2020 | Nov 15 | Nov 15 | 1 | 1 |  |  | 150.61 | 147.30 | 150.61 |
|  |  | BNC16 |  | Nov 14 | Nov 14 | 1 | 1 |  |  | 84.82 | 75.21 | 84.82 |
|  |  | BNC20 |  | Nov 16 | Nov 16 | 1 | 1 |  |  | 99.29 | 95.71 | 99.29 |
|  |  | BNC41 |  | Nov 21 | Nov 21 | 1 | 1 |  |  | 86.58 | 84.45 | 86.58 |
|  |  | BNC42 |  | Nov 23 | Nov 23 | 1 | 1 |  |  | 89.32 | 88.69 | 89.32 |
|  |  | BNC28 | 2021 | Nov 15 | Nov 15 | 1 | 1 |  |  | 98.74 | 98.52 | 98.74 |
|  |  | BNC29 |  | Nov 16 | Nov 16 | 1 | 1 |  |  | 111.82 | 107.57 | 111.82 |
|  |  | BNC30 |  | Nov 15 | Nov 15 | 1 | 1 |  |  | 83.77 | 83.24 | 83.77 |
| **W** | ⑪ | BNC18 | 2020 | Nov 23 | Nov 23 | 1 | 1 |  |  | 113.77 | 108.39 | 113.77 |
|  |  | BNC19 |  | Nov 23 | Nov 23 | 1 | 1 |  |  | 132.40 | 105.04 | 132.40 |
|  |  | BNC43 |  | Nov 23 | Nov 23 | 1 | 1 |  |  | 112.24 | 108.38 | 112.24 |
|  |  | BNC44 |  | Oct 30 | Oct 30 | 1 | 1 |  |  | 123.02 | 116.32 | 123.02 |
|  |  | BNC31 | 2021 | Nov 15 | Nov 16 | 2 | 2 |  | 1 | 129.32 | 116.20 | 64.66 |
|  |  | BNC32 |  | Nov 15 | Nov 16 | 2 | 2 |  | 1 | 129.56 | 116.23 | 64.78 |
| ⑫ **Sedentary** | | BNC17 | 2020 |  |  |  |  |  |  |  | 9.43 |  |
|  |  | BNC45 |  |  |  |  |  |  |  |  | 5.18 |  |
|  |  | BNC27 | 2021 |  |  |  |  |  |  |  | 0.53 |  |
